# The Many Faces of Indeterminacy in Interactive Deadbots

**DOI:** 10.1007/s13347-026-01089-2

**Published:** 2026-04-13

**Authors:** Atay Kozlovski, Edina Harbinja, Roel Dobbe

**Affiliations:** 1 TU Delft, Delft, The Netherlands; 2 Birmingham Law School, University of Birmingham, Birmingham, UK

**Keywords:** Digital afterlife, Digital immortality, Digital resurrection, Interactive deadbots, Griefbots, Indeterminacy

## Abstract

Advances in generative AI have given rise to a growing industry centred on interactive representations of deceased individuals. Within this emerging “digital afterlife industry”, *interactive deadbots* (IDBs) are presented as hyper-realistic avatars that use a person’s likeness, voice, and personal data to simulate conversational interactions with them. Rapidly moving from a niche experiment to a mainstream phenomenon, IDBs are poised to reshape the ethical, social, legal, and governance landscapes surrounding death, mourning, and digital legacy. This paper examines the disruptive nature of IDB technology through a multidisciplinary lens, using the concept of indeterminacy as its guiding analytical framework and a novel way to conceptualise the unstable field. Rather than advancing a unified understanding of indeterminacy, we introduce a structured analytical map and provisional taxonomy that distinguishes technological, social, philosophical, legal, and regulatory manifestations of indeterminacy in IDBs. By offering a tentative and necessarily selective map of this fluid and nascent field, we explore how indeterminacy and IDBs intersect. The paper examines how IDBs amplify existing forms of indeterminacy and how indeterminacy itself shapes the development and use of these systems across five domains: technological, social, philosophical, legal, and regulatory.

## Introduction

Recent developments in machine-learning methods and large language model (LLM) capabilities have given rise to a burgeoning industry focused on the digital afterlife and the possibility of maintaining, through simulation, some form of connection and communication with the dead. These generative AI–powered systems, trained on large amounts of personal data, present as hyper-realistic digital avatars that reproduce the likeness, voice, behaviour, beliefs, and other personal traits of a specific person. Unlike earlier technologies, interactive deadbots (IDBs) differ from static digital memorials, such as pre-recorded memory bots or commemorative profiles, in that they enable real-time interaction with a simulation of the deceased and are being deployed for a wide range of purposes: grief support, legacy preservation, educational initiatives, the continuation of personal bonds, and more.

While companies offering IDB-related services (e.g., StoryFile, HereAfter AI, 2wai) portray their products as overwhelmingly beneficial, the reality is far more complex and filled with uncertainty. Do IDBs actually support users in coping with grief? Can an IDB meaningfully simulate the nuances of a person’s personality? What impacts might prolonged human–IDB engagements have on users’ mental health, well-being, and social relationships? How might IDBs reshape norms surrounding death? And do existing laws and regulations offer protection either to the users of these technologies or to the individuals being digitally simulated?

These questions do not arise merely because IDBs are new or technologically sophisticated; rather, they point to deeper conceptual challenges that complicate any attempt to evaluate them. Some of these challenges reflect the fact that IDBs inhabit the domain of death, memory, and the deceased, where ethical, social, regulatory, and legal frameworks are already defined by longstanding ambiguities and contested assumptions, which in themselves make the role and function of IDBs difficult to assess. Others arise because IDBs actively unsettle and disrupt these very domains, blurring established categories and norms in ways that introduce additional interpretive and practical difficulties. Taken together, these challenges reveal the broader theme of indeterminacy, which is at the heart of this paper, and, as we argue below, shapes the domains in which IDBs are situated as well as the new disruptions they bring about. We do not treat indeterminacy as inherently beneficial or pathological; rather, we employ it as an analytical lens to illuminate how its various manifestations arise within IDBs and shape their social, legal, philosophical and regulatory implications.

This paper offers a collaborative, interdisciplinary examination of the many different “faces” in which indeterminacy arises in connection with IDBs. Drawing on the emerging literature in the field, we highlight areas of conceptual density and zones of intellectual scarcity, and offer the first structured attempt to chart the indeterminacy inherent in IDB technologies. Our contribution lies less in redefining indeterminacy as a concept, and more in demonstrating how its different forms are provoked, intensified, and industrialised by IDBs as a socio-technical phenomenon situated in the culturally and normatively charged terrain of death. We proceed from the premise that the field is nascent and unsettled, and therefore our contribution is necessarily provisional and selective rather than exhaustive.

The paper is structured as follows. Section 2 reviews existing real-world IDB use cases, introduces a framework for defining key terms in this space, and concludes with a single hypothetical case study that serves as a reference point throughout the paper. Section 3 focuses on the notion of indeterminacy, situates it within relevant interdisciplinary literature, and clarifies how we use this term in each of the five domains that we discuss. Section 4, which forms the core of the paper, examines how IDBs intersect with indeterminacy in five domains: technological, social, philosophical, legal, and regulatory. Section 5 concludes.

## IDBs, Definitions, and Case Study

This section begins by providing a general overview of ways in which AI is currently being used to simulate the dead by different private and commercial entities. We then briefly discuss a terminological challenge that exists in the discourse revolving around this emerging technology and clarify the terms we will use throughout this paper. Finally, we end this section by describing a hypothetical use case which will serve as a reference point for our analysis throughout the paper.

### Interacting with the Dead

While this paper is not intended as a comprehensive review of the emerging field of ‘digital afterlife’, we believe it is essential to provide some insight into this rapidly expanding industry and domain of research. As such, to provide a brief overview, it will be easiest to look at the two extreme edges of the spectrum of AI-based technology used to simulate the dead. At one end of the spectrum are capture-first, interview-led memory platforms that store stories, voices, and images for later interactive playback. HereAfter AI, for instance, offers a subscription model through which people narrate life stories and attach photographs; family members can then “converse” with a memory-avatar that simply retrieves this corpus (HereAfter AI, 2024–2025)^1^. *StoryFile* takes a similar capture-first approach in video: individuals record answers across a topic “StoryLine”, and viewers later ask questions to which the system selects the best pre-recorded reply, “video that talks back”, with the consumer app currently on a waitlist while enterprise tools remain active (StoryFile, 2024–2025)^2^. These systems feel interactive but are essentially retrieval-driven, not free-form; they index and surface what was actually said.

At the other end lie LLM-powered tools that simulate new utterances “in the voice” or style of a specific deceased person. *Project December*, one of the first publicly discussed platforms, presents itself explicitly as a way to “simulate the dead”: a user supplies traits and snippets, and a large model generates open-ended conversations; mainstream reporting shows how this has evolved from an art and personal experiment to a grief use-case (Milmo, 2024). *You*,* Only Virtual* markets “versonas” that text or call, promising adaptive and ongoing presence (YOV, 2025)^3^. Services such as *Seance AI* package a short session with a GPT-class model as a memorial interaction (Seance AI, 2023–2024)^4^. In some cases, these technologies have been deployed in striking public settings. In Arizona, for example, the family of Chris Pelkey, killed in a 2021 road-rage shooting, created an AI rendering of him to deliver a victim impact statement in court, telling the defendant, “I believe in forgiveness”, arguably the first appearance of a digitally simulated person in a legal proceeding (Neff, 2025). In this instance, however, the avatar reportedly delivered a script written by Pelkey’s sister rather than generating the statement autonomously (Pereira, 2025). Similarly, journalist Jim Acosta recently interviewed a generative avatar of Parkland shooting victim Joaquin Oliver, created with his parents’ support. In contrast, parents of other victims launched “The Shotline”, a campaign using AI voices of the deceased to lobby Congress on gun reform (Kerr, 2025).^5^

### Definitions

The previous section provided a brief overview of the many and varied uses of Interactive Deadbots (IDBs). Given this diversity, it is perhaps unsurprising, but nevertheless troubling, that the literature has yet to converge on agreed-upon terminology for this technology. Despite the growing number of use cases and applications, neither popular nor academic discourse has developed a stable vocabulary to describe these phenomena.

To illustrate this terminological fragmentation, a wide range of terms have been used to describe similar and often overlapping systems, including “thanabots” (Henrickson, 2023), digital duplicates (Danaher & Nyholm, 2024; Kozlovski, 2025), digital immortality (Brown, 2017), “post-mortem avatars” or “deadbots” (Hollanek & Nowaczyk-Basińska, 2024), digital resurrection (Leaver, 2019), “griefbots” (Jiménez-Alonso & Brescó de Luna, 2022), “ghostbots” (Figueroa-Torres, 2024; Harbinja et al., 2023), “mind clones” (Voinea et al., 2025), “generative ghosts” (Morris & Brubaker, 2024), “digital zombies” (Basset, 2015), AI twins, the digital afterlife industry (Öhman & Floridi, 2017), and Interactive Personality Constructs of the Dead (IPCDs; Stokes, 2025), among others.

To help make sense of this overabundance of terms and clarify how we will use terminology throughout this paper, we propose a conceptual framework that distinguishes between different levels of analysis. The framework consists of four levels: (1) the field of analysis, (2) the modality within that field, (3) the type of artefact according to use or function, and (4) the specific instance of that type.

The first level of the framework represents the highest level of abstraction and includes umbrella terms such as Digital Afterlife or Digital Immortality. This field-level classification is particularly important for distinguishing between AI systems that simulate the living^6^ and those that simulate or represent the dead. Such terms encompass the broader socio-technical domain concerned with preserving, curating, governing, and in some cases simulating a person’s presence after death (Basset, 2015; Jacobsen, 2017; Öhman & Floridi, 2017; Savin-Baden & Mason-Robbie, 2020).

The second level of classification - modality - refers to the way in which the presence of the deceased is mediated or engaged with. Modalities capture how posthumous presence is made available, rather than why or for what purpose. Examples include archival modalities, static representational modalities, interactive simulations, and immersive or embodied experiences. The focus of this paper is on the modality of Interactive Deadbots (IDBs) and the various types and instances that fall within it.

The third level of the framework concerns types, which differentiate artefacts within a given modality according to their intended use or primary function. Within the modality of IDBs, for example, one can identify educational IDBs designed to simulate interactions with historical figures for learning purposes; relational IDBs intended to sustain or extend interpersonal relationships with the deceased; griefbots created specifically to assist individuals in coping with loss; legacy avatars designed to continue aspects of a person’s life’s work; and political avatars deployed to promote specific ideologies or agendas.

Finally, the fourth level of the framework refers to specific instances of a given type. Examples include the testimonial avatar of Christopher Pelkey (Neff, 2025) or Jessica’s griefbot created using Project December (Fagone, 2021).

### An IDB of Anne Clark - Fictional Case Study

With the aid of this new conceptual framework, we can more effectively explain the focus of this paper. Our main goal is to highlight various forms of indeterminacy that arise from the emerging use of IDBs. However, as the previous section demonstrated, there are multiple types of IDBs and numerous instances within each type. To make our analysis more precise, we therefore narrow our focus to a single hypothetical, yet in our view plausible and imminent, type and instance of IDB that we can refer to throughout the paper:

Anne Clark passed away in 2040, having spent her final years contributing journals, recordings, and extended interviews to a project intended to create an interactive digital version of herself for her family. The resulting “Anne Clark IDB” generates new, turn-by-turn replies in her familiar tone and style, first via text, later via a synthesised voice, and eventually via a video avatar explicitly crafted to resemble her. It is primarily used by her family to continue hearing her stories, asking questions, and maintaining a sense of connection. At the same time, the system is technically compatible with major social platforms, making it possible, if her family chooses, to interact with “Anne” in shared digital spaces such as group chats, memorial pages, or private community networks, offering both an intimate domestic presence and the potential for more social forms of interaction long after her death.^7^

Anchoring the paper on this example will allow us to concretely illustrate and evaluate the many faces of indeterminacy associated with emerging IDB technologies.

## Indeterminacy

This section discusses how we use the notion of indeterminacy and, in doing so, lay the groundwork for the analysis that follows. To clarify our focus, it is helpful to begin by stating what this paper does not seek to do. This is not a conceptual paper aimed at developing a unified or comprehensive account of indeterminacy, nor does it adopt a single conceptualisation of indeterminacy and apply it throughout. Instead, indeterminacy is used as a heuristic lens to highlight central challenges raised by IDB technologies from five separate perspectives: technological, social, philosophical, legal, and regulatory. Our aim is deliberately synoptic: to map challenges, draw together dispersed strands of scholarship, and identify where IDBs generate novel forms of indeterminacy and where existing indeterminacies constrain their design, deployment, and evaluation. Each section presents key issues within its domain, not exhaustively, but in order to provide an overview that conveys the full breadth and scope of the phenomenon.

In what follows, we present how indeterminacy is understood in each of the five perspectives, together with the main issues discussed in each section:

1. The technological perspective focuses on the nondeterministic nature of LLM-powered systems and how it impacts the ability to predict or explain an IDB’s outputs. The fact that these systems incorporate biases, produce hallucinations, and lack explainability and transparency (‘black box’) poses significant challenges for how users engage with them, how designers approach their creation, and how governance schemes can be developed.
2. The social perspective examines how IDB technologies intersect with cultural indeterminacy, that is, the fluidity, uncertainty, and plurality that underlie social and cultural norms. In this context, we explore three key intersections: grief trajectories, relationships with the dead, and power dynamics in memorialisation practices.
3. The philosophical perspective addresses three domains in which IDBs intersect with indeterminacy: metaphysical, concerning the relational status between an IDB and the represented individual; epistemological, concerning user interactions with IDBs and the nature of the knowledge such interactions generate; and rational, concerning the extent to which an IDB can be aligned with a particular individual’s values and preferences.
4. The legal perspective applies the lens of moderate legal indeterminacy (whereby many straightforward cases have clear legal answers, some, particularly those at the margins of rules or involving complex fact patterns, admit multiple outcomes; Hart, 1961) to IDBs in three areas: (1) status and harm, i.e. postmortem law’s unresolved person/thing boundary and the question of who can be harmed; (2) postmortem privacy and dignity, and (3) liability and accountability under emerging AI laws, consumer protection law, and product safety/liability legal frameworks.
5. The regulatory perspective focuses on the difficulty of constructing a governance environment that is fit for purpose for IDBs in conditions of rapid technological change and contested social meaning, especially in death and bereavement contexts. It emphasises how IDBs strain prevailing regulatory categories and logics (notably decentred, risk-based and sector-siloed approaches), while raising legitimacy questions about who should set standards, how vulnerability and manipulation are defined, and how accountability is allocated across developers, platforms, and intermediaries (Table 1).

**Table 1:** Overview

Perspective	Indeterminacy Type	Focus
Technological	Nondeterministic (stochastic outputs; opacity; hallucinations/bias)	Predictability/controllability of outputs; explainability; design and governance implications
Social	Cultural indeterminacy - fluidity/ambiguity of practices and meanings	Grief trajectories; relationships with the dead; power dynamics
Philosophical	Metaphysical, epistemic and rational indeterminacy	Relation between individuals and their IDBs, reliability of Human-IDB interactions, Human-IDB alignment
Legal	Structural legal indeterminacy (moderate; open texture; plural values)	Status and harm; post-mortem privacy/dignity; liability and accountability
Regulatory	Fragmentation and discrepancy across regimes (overlap/gaps; forum allocation)	Boundary problems between AI, data, consumer, safety/liability, platform governance, enforcement capacity and “fit for purpose”

## Indeterminacy and IDBs

This section examines the various manifestations of indeterminacy related to or caused by IDB technology. While the phenomenon is fundamentally socio-technical, analytical clarity requires its consideration through distinct yet interrelated perspectives: technological, social, philosophical, legal, and regulatory. Each dimension reveals a particular modality of openness, contestability, or under-determination, whether in technical design, social meaning, normative reasoning, or institutional control. In each of the following sections, we will highlight both how IDBs exacerbate existing or introduce novel forms of indeterminacy and how indeterminacy in a given domain constrains the design, use, or evaluation of IDBs. The discussions in each section are by no means exhaustive, rather they are intended to highlight key issues and lay the groundwork for future in-depth research into each category.

### The Technological Face of Indeterminacy

We begin our analysis of the different faces of indeterminacy with the technological perspective as it intersects most directly with the four other perspectives discussed below. This section addresses two primary issues: first, the nondeterministic nature of LLM-powered IDBs; and second, three features of LLMs that play a central role in generating indeterminacy across the other perspectives: biases, hallucinations, and lack of transparency.

####  IDBs and Nondeterminism

From a computer science point of view, IDBs, like many other ‘AI’ systems, particularly those powered by LLMs, should be regarded as nondeterministic by nature. This is perhaps best understood by looking at the contrast with determinism. A ‘deterministic system’ is one in which no randomness influences the development of future states; given the same input, it will always produce the same output and follow the same sequence of internal states. For instance, while the previous generations of rule-based or non-generative chatbots could produce a wide range of content, the system’s responses were predictable and pre-determined by the designers (Caldarini et al., 2022). By contrast, LLM-powered IDBs operate in inherently stochastic ways, meaning their behaviour can vary even under identical conditions, making IDBs inherently ‘nondeterministic systems’ (Atil et al., 2025).

Importantly, this inherent nondeterminism should not be mistaken for complete randomness. In practice, many mechanisms are implemented to ensure that an IDB’s outputs remain coherent, consistent, and contextually appropriate. First, IDBs are typically powered by a base LLM which provides the capacity to generate fluent natural language (Brown et al., 2020). On top of this foundation, fine-tuning or integrating a curated knowledge base enables the IDB to produce outputs that are relevant to a particular domain or tailored to emulate a specific individual’s style (Ouyang et al., 2022). For instance, an IDB of Anne Clark would draw on her personal data to generate conversational interactions reflecting her past experiences, knowledge, and manner of speaking. Finally, additional layers of control, such as guardrails that restrict certain topics, words, or behaviours, are often implemented to ensure the IDB operates within acceptable boundaries. In practice, these controls include safety classifiers, prompt/response filters, constrained decoding, and retrieval-augmented generation (RAG) with provenance pointers (Weidinger et al., 2021; Holtzman et al., 2020; Lewis et al., 2020); they improve plausibility and local fidelity but still do not yield a deterministic implementation.

#### LLM Features and Indeterminacy

Alongside the nondeterministic nature of LLM-powered IDBs, the LLM architecture itself brings with it certain well-known problematic features, which directly contribute to the emergence of indeterminacy: bias, hallucinations, and lack of transparency.

Starting with bias, while this term can simply refer to a system’s tendency to gravitate toward certain types of outputs, a necessary trait for instance if we are trying to ‘personalize’ or ‘align’ a system to respond in a way that realistically simulates Anne, the term is usually reserved for cases where this tendency is unintended or undesirable (e.g. prioritizing certain expressions or causing discriminatory outcomes). Thus, we say that a recommendation system is ‘aligned’ if it produces fair hiring recommendations according to our definition of fairness, and biased if it produces discriminatory recommendations. We further expand on this issue in Sect. 4.3.2.

At a technological level, bias can be best seen as a function of abstraction. Abstraction is a general principle of software development, defined by Ousterhout as “a simplified view of an entity, which omits unimportant details” (Ousterhout, 2021). It is exactly in the inherent omission of details that opportunities for bias are introduced, as in the statistical definition, bias means “a systematic distortion of a statistical result due to a factor not allowed for in its derivation” (Oxford Dictionary). Here we refer to undesirable bias as emerging from a misabstraction (noun), which we understand as “a representation of an entity, phenomenon, or procedure that omits critical contextual information and renders that representation problematic when it is reintegrated into the context of the sociotechnical system for which it has been made” (de Troya et al., 2025). As such, bias and abstraction are interrelated and context-dependent, requiring a sociotechnical frame for analysis and design of algorithmic artefacts (Selbst et al., 2019). Relatedly, the processes of abstraction and representation within IDBs depend not only on the quality and representativeness of the available data (pre-existing bias), but also on how specific system design choices are made (technical bias), and how the system is ultimately recontextualised and used in practice (emergent bias) (Dobbe et al., 2018).

Turning to the issue of hallucinations, while outputs of IDBs depend fundamentally on the quality and representativeness of the data used to train either the underlying LLM or the subsequent components in its control layers (Birhane et al., 2023), even when grounded in a personal and large corpus, such models typically interpolate and extrapolate beyond the available data (Morris & Brubaker, 2025). The term Hallucination is used to refer to such cases in which the model, or in our case, an IDB, produces a factually incorrect response but in convincing and confident language. This is perhaps the most widely recognized technical limitation of LLMs, and increasingly, researchers have argued that hallucinations are not simply bugs that can be fixed, but rather inherent features (Dumit and Roepstroff 2025) of LLMs, an issue that can be mitigated but not fully eliminated. Several well-documented cases illustrate this problem. For example, Google’s “AI Overviews” has produced outputs ranging from the absurd to the dangerous, such as advising users to “eat at least one small rock per day” or suggesting adding “non-toxic glue” to add tackiness to pizza sauce (McMahon, 2024).

Finally, the issue of transparency, or inscrutability of LLM-based systems (Kroll, 2018), means that one typically cannot explain which sources of information underpin a given response - often referred to as the ‘Black Box’ problem (Burrell, 2016). The origins of an IDB’s responses are often unknowable, leaving users uncertain as to whether they can, or should, trust any given output (Birhane et al., 2023). And while RAG and citation-style justifications may enhance epistemic transparency, users still cannot know whether this is what Anne would have said, only that the answer provided by the system is at best textually supported. As such, even with these many mechanisms designed to minimise randomness, increase transparency, and ensure that IDBs produce relevant and coherent outputs, the inherent indeterminacy of such systems prevents any guarantee of accuracy - a problem for any users of such systems but which carries particular social and emotional weight in bereavement contexts (Henrickson, 2023; Harbinja, Edwads & McVey 2023) an issue we further discuss in Sect. 4.2.

Technological indeterminacy, therefore, should not be viewed as a temporary engineering limitation but as a structural feature of generative, sociotechnical systems (Dobbe et al., 2021). In the following sections, we examine how IDBs and these technical characteristics give rise to distinct forms of indeterminacy and raise broader concerns from social, philosophical, legal, and regulatory perspectives, each explored in a more nuanced and contextualised manner.

### The Social Face of Indeterminacy

The social face of Indeterminacy in IDBs builds on discussions of the indeterminate nature of cultural and social norms (Gerber, 2016). This indeterminacy operates at the level of lived experiences and reflects, as John Dewey expressed it, “the vast range of things experienced in an indefinite variety of ways” (Dewey, 1981). Thus, like many other disruptive emerging technologies, IDBs surface in a domain filled with ambiguities and plurality of viewpoints. In our context, the indeterminacy relates to how IDBs challenge and disrupt social practices related to remembrance, agency, dignity, authorship of memory, and the governance of platforms that now mediate grief, intimacy and public history (Lindemann, 2022; Harbinja et al., 2023; Hollanek & Nowaczyk-Basińska, 2024; Morris & Brubaker 2025; McStay, 2024; Öhman, 2024, Kneese, 2023). In what follows, we discuss three such intersections: grief trajectories, social and relational ambiguities, and control of power dynamics.

#### Grief Trajectories

Contemporary bereavement research rejects the notion of grief as a linear process of detachment. Instead, it conceives of it as a dynamic and continuing bond, an ongoing relationship reorganised over time (Klass et al., 1996; Stroebe & Schut, 1999). IDBs intervene directly in this relational space. They simulate the voice, language and affect of the deceased, inviting communication that can sustain or reconfigure the relationship. For many users, this interaction provides solace or facilitates meaning-making (for example, helping them narrate the loss, express unresolved feelings, or integrate the deceased into an altered life story); for others, it reinforces avoidance, dependency or emotional displacement (for example, substituting bot interaction for real-world support, prolonging denial of the death, or displacing difficult emotions into repetitive “contact”) (Lindemann, 2022; Henrickson, 2023). The plurality of grief trajectories means that there is no single normative baseline for identifying ‘healthy’ mourning, or a clear demarcation between therapeutic use and harmful entanglement with IDBs (Krueger & Osler, 2022; Betancur & Maria, 2025).

This issue is intensified by synthetic presence. The more convincing an IDB becomes, the more difficult it is for users to maintain the cognitive boundary between simulation and reality (McStay, 2024). In such cases, even if transparency prompts or disclaimers are put in place to mitigate such aspects, they may not be sufficient if the interface continually conveys intimacy and apparent autonomy. When outputs imply agency, “Anne would have chosen…”, users risk being normatively guided rather than merely informed (Morris & Brubaker 2025). The outcome of these interactions are neither predictable nor uniform: some users regain stability (in the sense of returning to everyday functioning and gradually reducing reliance on the bot as acute grief recedes); others spiral into dependency (in the sense of escalating, compulsive use that displaces offline relationships and makes coping contingent on continued interaction) (Betancur & Maria, 2025).

These diverse grief trajectories reveal that the social meaning of IDB interaction is itself indeterminate, varying across users, contexts and temporal stages of grief. What is novel is not the cultural diversity of grief, but the way IDBs industrialise and algorithmically mediate that diversity through interactive synthetic presence, producing new feedback loops and boundary disputes (support vs. manipulation; memorialisation vs. commodification) that resist stabilisation by existing social norms.

#### Cultural Diversity in Relationships with the Dead

IDBs are embedded in social contexts marked by a high degree of indeterminacy, contexts that are culturally mediated and subject to change over time. At the individual level, ambiguities arise when considering the kinds of relationships people can form with IDBs. Many individuals develop strong emotional attachments to AI companions in general, and to IDBs in particular; yet it remains unclear whether such interactions can meaningfully constitute friendship or romantic partnership (Lott & Hasselberger, 2025), or whether these categories can coherently be applied to non-human entities at all. Beyond these categorical uncertainties, increased interaction with IDBs is likely to have profound implications for both individual users and society at large. For instance, will extensive engagement with IDBs alleviate or exacerbate loneliness (Alvarado, 2025)? Will IDBs transform, reconfigure, or further fragment how individuals cope with grief and death (Iglesias et.al. 2024)?

At a broader societal level, practices of grief and mourning change over time as burial customs, religious ceremonies, and traditions of remembrance evolve in form and meaning (Sumiala, 2012). These dynamics become even more complex when viewed from a global, cross-cultural perspective. Global platforms frequently export design defaults rooted in Western, English-language, and often male-coded imaginaries. These ostensibly “neutral” templates risk flattening (Anderson et al., 2024) the diverse ways in which societies honour, remember, or relate to their dead (Figueroa-Torres, 2024; McStay, 2024). For some communities, maintaining communication with the deceased may align with established ritual or spiritual traditions; for others, it may border on sacrilege. The same interface can therefore appear meaningful or even healing in one cultural context while being experienced as profoundly inappropriate in another.

Cultural pluralism thus plays a decisive role in shaping how death, mourning, and posthumous representation are understood, as well as how governance frameworks surrounding these practices are constructed. Yet these dimensions remain underexplored in existing broader debates on technology governance, which often overlook the cultural specificity of death-related norms and values (ÓhÉigeartaigh et al., 2020; Silverman et al., 2021; Alsaleh, 2024).

#### Control and Power Dynamics

Who controls or owns an IDB? Platforms and providers govern the infrastructure, models, and data pipelines; families and communities hold affective and, in some jurisdictions, limited legal stakes; and estates may assert intellectual-property or publicity rights. Yet the preferences of the deceased—whether they wished to be digitally revived, and if so, how and by whom—are often unknown or contested (Haneman, 2025; Harbinja et al., 2023; Jurcys et al., 2024). Not to mention that the notion of control over AI systems itself spans technical, emotional, and moral dimensions, and is highly susceptible to a form of conceptual engineering (Köhler et al., 2025). In response, some scholars have advocated for a shift from ownership to stewardship, an approach embedding duties of care, proportionality, auditability, and sunset clauses; enabling participatory governance and consent-by-proxy; and balancing memorial freedom with relational accountability (McStay, 2024; Haneman, 2025). Still, the question of who should act as steward remains unresolved. Families, estates, communities, and public institutions such as archives or museums each embody different power dynamics and competing visions of whose memory counts.

Meanwhile, platforms have become de facto co-authors of the past. Through algorithmic curation, moderation, and recommendation systems, they impact which versions of the dead remain visible and which fade (Morris & Brubaker, 2025). This dynamic exemplifies what Öhman and Floridi (2017) call the Digital Afterlife Industry, a political economy of memory governed by engagement metrics and proprietary design. In this model, remembrance itself becomes a kind of commodity: “affection-as-a-service” links grief support to purchasing power, while terms of service dictate the conditions of mourning (Figueroa-Torres, 2024). Platform ownership also renders collective memory precarious. Policy shifts, API changes, or algorithmic updates can erase archives, distort provenance, or rewrite narratives (Öhman, 2024; Kneese, 2023).

Social indeterminacy is thus not incidental but structural. By entwining personal grief, cultural pluralism, and platform governance, IDBs blur the boundaries between intimacy, representation, and control—revealing social meaning as co-produced among designers and users, the living and the dead, local traditions and global infrastructures.

### The Philosophical Face of Indeterminacy

The creation and proliferation of IDB technology raises a great number of profound philosophical questions - epistemological, phenomenological (Stokes, 2025), metaphysical, relational (Campbell et al., 2025), and others. In this section, we start by looking at how IDBs introduce new and exacerbate existing forms of indeterminacy from both metaphysical and epistemological perspectives. We then discuss how indeterminacy in rational decision-making poses an inherent challenge to the possibility of Human-IDB alignment.

####  Metaphysical and Epistemological Indeterminacies in IDBs

Starting with a metaphysical concern, significant uncertainty surrounds the nature of the relation between an IDB and the person it represents: does the IDB of Anne Clark act as her proxy after death (Sweeney, 2023), constitute an extension of her personhood (Karpus & Strasser, 2025), or extend her ‘person-span’ (Iglesias et al., 2024)? Moreover, there may be indeterminacy within these very categories. Karpus and Strasser (2025), for instance, adopt a Parfitian theory of psychological connectedness (Parfit, 1984) to evaluate whether an IDB should be regarded as an extension of one’s personhood. Their account allows for degrees of connectedness, thereby introducing indeterminacies reminiscent of the sorites paradox (Raffman & Hyde, 2025): “there will be cases where the question of whether there is a continuation of a person will, as a matter of fact, not have a categorical answer” (Karpus & Strasser, 2025, p. 16). It remains unclear which, if any, of these interpretations is correct, whether they are perhaps mutually compatible, and who, if anyone, has the normative authority to determine how such digital continuations ought to be understood.

Turning to the epistemological challenges linking IDBs and indeterminacy, this is an area in which the technological features of LLM-powered systems discussed in Sect. 4.1 become particularly salient. Epistemology concerns how knowledge and beliefs are formed and justified, rendering issues such as bias, opaque training data, and hallucinations especially pressing in this context. Recent literature highlights growing concern about the epistemic risks that arise in human–IDB interactions. For example, Coeckelbergh’s analysis of AI in social media - focusing on the manipulation of beliefs, the formation of epistemic bubbles, and reliance on statistical rather than contextual forms of knowledge - applies closely to IDBs. Similarly, Schneider’s (2025) work on “chatbot epistemology” questions whether information produced by such systems can legitimately function as justification for belief. When combined with well-documented tendencies toward automation bias (Kozlovski, 2026) and anthropomorphism (Janson, 2023), IDBs create fertile ground for epistemic indeterminacy. Users may reasonably ask: Can I rely on the IDB’s output? Is its advice trustworthy? Would my loved one have actually said this?

These questions connect directly to the discussion in Sect. 4.2.2 on relationships with IDBs as a social phenomenon and illustrate that the disruptive character of this technology exceeds the typical uncertainty associated with novel technological developments. Rather, IDBs may constitute a paradigm shift comparable to the advent of social media—one that challenges established forms of human–human interaction, prevailing modes of knowledge acquisition, and, ultimately, aspects of the social and political order.

#### Rational Indeterminacy and Human-IDB Alignment

If IDBs are intended to simulate interactions with individuals in open-ended conversations, they must be able to capture not only external traits, such as appearance, mannerisms, voice, tone, word choice, etc., but also aspects of the target individual’s inner self, including their preferences, values, beliefs, and attitudes. Successfully capturing and simulating this full range of characteristics can be framed as an ‘alignment problem’ (Christian, 2020; Gabriel, 2020) that IDBs must address. And while there are extensive technological discussions regarding methods and strategies for alignment (Russell, 2019; Sarkar, 2025), here we approach the issue from a philosophical perspective, highlighting a fundamental challenge related to the existence of widespread indeterminacy in rational decision making.

To begin, we understand the relevant notion of rational decision making as denoting the process by which values and reasons determine the all-things-considered course of action one ought to take in a given situation (Ullmann-Margalit & Morgenbesser, 1977; Chang, 2016). We understand indeterminacy in rational decisions as referring to cases in which no clear-cut conclusion as to which course of action ought to be taken can be arrived at even when all relevant information is known. Such situations are best characterised by what Joseph Raz termed ‘The Basic Belief’:

“that most of the time people have a variety of options such that it would accord with reason for them to choose any one of them and it would not be against reason to avoid any of them” (Raz, 1999, p. 100).

Importantly, scholars have argued that cases of rational indeterminacy are both widespread and recurrent across a range of normative decision-making contexts - high-stakes and low-stakes, individual and collective - and across diverse domains, including healthcare choices, career decisions, legal adjudication, technological design, and more (Broome, 1997; Chang, 2002; Kozlovski, 2022). While decisions under such conditions may be experienced as difficult, insofar as the reasons supporting different options fail to decisively favour one over another, Ruth Chang has argued that it is precisely these cases that provide opportunities for agents to actively shape their rational identity: “It is the point at which we come into our own as self-governing agents” (Chang, 2016, p. 19).

Assuming this form of rational indeterminacy is indeed widespread, the implications for the design and function of IDBs are far-reaching, as it suggests an inherent limitation to human–IDB alignment. In cases of rational indeterminacy, human agents are not guided toward a uniquely correct response but must instead choose among multiple options consistent with their values and reasons. When an IDB encounters such a case and resolves it in favour of one option, the resulting output reflects a choice made by the system rather than a determination grounded in the represented individual’s own rational agency.

Applied to the case of an IDB representing Anne Clark, this observation leads to two conclusions. First, when faced with instances of rational indeterminacy, Anne’s IDB can at best simulate actions or speech acts that Anne might have performed. While this may appear trivial, simulations are, after all, by definition not the real thing; it stands in tension with the imaginaries and marketing claims commonly advanced by IDB developers, which often suggest that such systems act as Anne herself would have acted. Second, whenever an IDB repeatedly resolves cases of rational indeterminacy by selecting among plausible options, it risks drifting over time (Patchipala, 2023; Ravindran, 2025; Zhi-Xuan et al., 2025) away from the values, dispositions, or patterns initially attributed to the represented individual.

To be clear, we do not claim that either predictability or drift pose insurmountable technical challenges. Rather, their significance lies in how they shape our understanding of the nature of IDB outputs and the persistent gap between those outputs and the actions of a rational human agent. Once this gap is acknowledged, designers cannot avoid addressing it, either explicitly or by omission. Should Anne’s IDB produce consistent outputs when faced with cases of rational underdetermination, or should it vary among plausible responses? Should it evolve over time by incorporating past system choices, or should it continually revert to an original behavioural profile? While these design decisions may themselves be subject to rational indeterminacy, how they are resolved will have significant implications for user interaction, trust, and the perceived metaphysical relationship between humans and their IDBs.

### The Legal Face of Indeterminacy

In legal theory, indeterminacy is conceptualised as a structural feature of legal reasoning, in which authoritative sources and interpretive methods permit multiple, equally plausible outcomes, thereby distinguishing it from mere uncertainty or ambiguity (e.g., Dworkin, 1986; Kress, 1987, 1989; Kennedy, 1976; Unger, 1983; Endicott, 2000). Unlike temporary uncertainty, indeterminacy is intrinsic to law, arising from linguistic ‘open texture’, vagueness, and shifting social contexts. (Endicott, 2000). Legal theorists distinguish between moderate indeterminacy, in which marginal or complex cases lack a single clear answer (Dworkin, 1986; Hart, 1961), and radical indeterminacy, which holds that the law is pervasively unsettled, allowing multiple legally justifiable outcomes in most cases (Kennedy, 1976; Unger, 1983). While well theorised in legal scholarship, its application to emerging technologies and to IDBs reveals novel dynamics that have received little scholarly attention (Calo, 2019; Mignanelli, 2024). This section adopts the lens of moderate legal indeterminacy and examines how IDBs, as novel and complex cases, intersect with and illuminate that indeterminacy. To do so, the analysis focuses primarily on the legal frameworks of the UK and the EU, with selective comparisons to US law where they serve to illuminate specific normative or doctrinal tensions.

#### Person vs. Things, Harm to the Dead

Post-mortem law has long occupied a complex terrain between persons and things. Legal systems recognise the corpse as neither fully subject nor object, the dead as neither rights-holder nor nullity (Cantor, 2010; Harbinja, 2022). Doctrines such as quasi-property (in common law, see Larson v. Chase, 47 Minn. 307 (1891); or Newman v. Sathyavaglswaran, 287 F.3d 786 (9th Cir. 2002) for a US perspective or Williams v. Williams (1882) 20 Ch D 659 and R v. Kelly [1999] QB 621) and moral or familial duties of respect (in civil law, see Carbonnier, 2004; or Böckenförde, 1991) attempt to stabilise this liminality. IDBs reuse traces of the deceased, and therefore unsettle categories: is the IDB a work (intellectual property), a dataset (data protection), or an extension of personality (dignity and privacy)?

The question of who can be harmed by an IDB, the deceased, the bot, the living, or society, maps onto unstable doctrinal foundations. Harms to the deceased confront the principle that rights die with the person (Actio personalis moritur cum persona, see Williams v Williams (1882) 20 Ch D 659 for a UK approach or Schuyler v. Curtis (1895) 147 N.Y. 434 for the US). Harms to the IDB presume legal subjectivity for a bot that does not exist. Harms to the living (e.g., emotional distress or reputational injury) may be recognised under Article 8 ECHR (Putistin v Ukraine) or tort law, but only derivatively and inconsistently. As Haneman (2025) and Harbinja (2022) observe, this fragmentation reflects the absence of a coherent postmortem framework. Harm is often legally invisible unless it can be translated into a protected interest of the living, and absent that translation, comparable injuries fall outside recognition.

Moreover, the emergence of IDBs introduces hybrid harms that existing categories cannot easily accommodate. As demonstrated in Sect. 4.2, psychological harms, prolonged grief, attachment, or distress, intertwine with dignitary and informational injuries. An IDB mimicking Anne Clark may simultaneously soothe and destabilise. McStay’s (2024) “presence effects” and Figueroa-Torres’s (2024) “affection-as-a-service” highlight how these products heighten emotional entanglement and commodify intimacy. The law’s apparatus, built for tangible damage, struggles to articulate such relational and affective injury.

#### Postmortem Privacy and Dignity

IDBs strain the normative coherence of post-mortem privacy and dignity. While data protection regimes generally cease at death, ethical and jurisprudential traditions affirm continuing moral duties (Edwards & Harbinja, 2013; Harbinja, 2017, 2020, 2022; Malgieri, 2018; Allen & Rothman, 2024), supported by empirical findings on post-mortem privacy perceptions (Harbinja, Morse & Edwards, 2025; Morse & Birnhack, 2020; Nakagawa & Orita, 2024). Without a legal subject, courts must route protection through the living. The Strasbourg Court has accepted that interfering with a deceased relative’s memory can violate the living’s Article 8 rights (Dzhugashvili v Russia 2010; Putistin v. Ukraine 2014), but this indirect recognition reinforces indeterminacy. In Anne’s case, a gap remains: the lawful reuse of her digital traces to generate speech she never made may be morally condemned but legally permissible where no derivative harm to the living meets established thresholds.

Jurisdictions diverge significantly: France’s Loi pour une République numérique permits digital-legacy directives; Italy allows heirs to exercise data-subject rights; the UK offers no statutory protection beyond contract. The US further fragments: some states, notably California (§ 3344.1 Civ Code (n.d.)) and New York (§ 50-f Civ Rights Law (n.d.)), recognise postmortem publicity rights, but only for public figures. Crucially, a distinct shift in US regulatory logic is emerging through ‘companion’ laws, such as California’s Companion Chatbot Law (SB 243) and New York’s General Business Law Article 47. Unlike replica laws that view synthetic identity as an appropriable asset, these rules treat the relationship-sustaining design, including ‘emotional prompting’ and ‘human-like responses’, as the locus of governance (GBS § 1700). While these initiatives move towards governing relational environments and recognise situational vulnerability, they focus solely on the living user; the deceased’s rights and IDBs remain fundamentally unarticulated.

This regulatory vacuum allows start-ups to model digital traces with little constraint. Developers may rely on platform terms or implied consent. Character.AI, for instance, allows characters modelled on the deceased under broad disclaimers. The boundary between homage, parody, and defamation remains a porous interpretive zone, allowing the creation of deadbots, from public figures like Joaquín Oliver (Kerr, 2025) to our Anne Clark, to persist without unequivocal prohibition. Related debates have also begun to emerge around digital replicas of living persons and whether individuals should possess a right to prevent AI-generated simulations of their identity, sometimes framed as a “right to remain dead” or a right against digital resurrection Haneman, 2025; Harbinja, Edwads & McVey 2023; Hollanek & Nowaczyk-Basińska, 2024; Harbinja, 2026).

#### Liability and Accountability

Legal indeterminacy shapes design through “anticipatory compliance” (Ortalda et al., 2025). Firms may use transparency labels or ethical affordances as liability governance to evidence reasonableness and reduce claims of foreseeable harm. In this sense, transparency functions as a defensive legal strategy: it is deployed to shape future assessments of duty, breach, and foreseeability in potential negligence or product-liability claims. The very vagueness of existing rules thus incentivises a patchwork of quasi-legal, self-regulatory mechanisms that operate as ex ante risk-management tools to anticipate potential liability.

Liability frameworks, which typically require an identifiable duty-bearer and a legally cognisable wrong, are also challenged by IDBs. IDBs disperse agency across platforms, developers, and users, thereby complicating accountability. It is rarely obvious who bears responsibility for emotional harm: the platform (EU Digital Services Act 2022/UK; Online Safety Act 2023), the developer (product liability laws, such as the EU Product Liability Directive 2024), or the user. This uncertainty over the applicable legal regime directly affects who can be held liable and under what standard. Whether an IDB’s speech is “illegal” or “merely offensive” remains unsettled, determining whether statutory notice-and-action obligations are triggered. If the conduct does not clearly qualify as unlawful content, platform duties may not apply, weakening accountability pathways. Under the EU AI Act 2024, conversational bots fall only under minimal transparency duties (Art 50), meaning that compliance may satisfy formal obligations without resolving deeper questions of harm, responsibility, or compensation. Thus, formal transparency compliance may operate as a liability shield, even where relational or psychological harms persist.

A further problem is the threshold for intervention regarding vulnerability. The EU AI Act 2024 prohibits AI that “materially distorts behaviour” by exploiting vulnerabilities linked to age, disability, or a “specific social or economic situation” (Art 5). Whether bereavement qualifies is an unresolved interpretive gap. While Calo (2018) notes digital architectures exploit situational vulnerabilities, the Act leaves mourning, an example of that, unmentioned. Rebrean and Malgieri (2024) argue for a fluid conception of vulnerability, which is not merely identity-based but also shaped by situational context and structural power imbalances that AI systems may amplify. On this view, grief becomes a legally salient condition, in which systems are engineered to sustain interaction. Until judicial precedent emerges, however, IDB behaviour oscillates between permissible entrepreneurship and prohibited manipulation.

Consumer and product safety frameworks are equally strained. Under the Unfair Commercial Practices Directive 2005 (UCPD) (and the UK Digital Markets, Competition and Consumers Act 2024-DMCCA), traders must not engage in aggressive practices toward the “especially vulnerable” (Art 5 UCPD; s.247(4) DMCCA). The Product Liability Directive 2024 defines “defect” through the safety a user is “entitled to expect”,(Art 7(1)). This standard is ill-suited for IDBs, where emotional dependency and presence effects defy conventional understandings of defect, damage, and risk. In English common law, tort law is also mostly unhelpful. While psychiatric harm is actionable and theoretically applicable to IDBs (Alcock [1991]; Mustapha v Culligan (2008)), the foreseeability of injury in grief-related interactions remains highly contested by the courts, as evidenced by the restrictive approach in Paul v Royal Wolverhampton NHS Trust [2024].

Ultimately, this indeterminacy is a constitutive feature of law’s engagement with disruptive technology.

### The Regulatory Face of Indeterminacy

While legal indeterminacy concerns the interpretive openness of rules, *regulatory indeterminacy* describes the institutional and epistemic conditions under which regulators, standards bodies, and firms must act despite incomplete knowledge and fragmented jurisdiction. As such, this subsection outlines the governance and regulatory disruptions posed by IDBs and situates these technologies within broader debates in regulatory theory about *disruption*, *harm*, *uncertainty*, and *indeterminacy*. We draw on Yeung’s (2008, 2017) typology of regulatory logics, Black’s (2002) notion of “decentred regulation”, Brownsword, Scotford, and Yeung’s (2017) three dimensions of disruption (legal disruption, regulatory disruption, and the challenge of constructing regulatory environments that are fit for purpose in light of technological disruption), and Ranchordás’s (2021) analysis of anticipatory governance. Like in the legal section, the analysis primarily interrogates UK and EU regulatory frameworks.

#### Category Indeterminacy

AI governance already operates within a complex architecture of overlapping horizontal and vertical regimes. Horizontal frameworks, such as the EU’s Artificial Intelligence Act (AIA), seek to regulate AI technologies across sectors through risk-based obligations. Vertical regimes, such as healthcare, media, consumer protection, and children’s safety, target specific domains (Yeung and Ranchordas, 2024). The coexistence of these layers produces what Smuha and Yeung (2025) term a *meta-regulatory* system: the state mandates internal compliance processes while delegating operational discretion to firms. The AIA exemplifies this “enforced self-regulation” model, requiring providers to implement and document risk-management systems that align with outcome-based legal objectives rather than prescribing substantive conduct ex ante.

For IDBs, this structure creates both flexibility and opacity. Most IDBs fall outside the AIA’s “high-risk” list (Annexe III), triggering only limited transparency duties (Arts 50–55). Yet their potential for psychological manipulation and emotional dependency suggests higher stakes than their regulatory classification implies. This discrepancy between risk potential and regulatory intensity reveals a first form of indeterminacy: category indeterminacy or the difficulty of fitting a socially and ethically complex artefact into predetermined regulatory tiers.

#### Decentred Regulation

Supervisory pluralism compounds the issue. At the EU level, the new AI Office coordinates enforcement, while each Member State must designate a competent authority (Art 70 AIA). Parallel regimes, the Digital Services Act 2022 (DSA), GDPR 2016, Copyright Directive 2019, govern related dimensions such as content moderation, data processing, and text-and-data mining. In the UK, where no single AI statute exists (Edwards, 2025), the Online Safety Act 2023 (OSA) anchors risk duties at the service level: Ofcom oversees user-to-user and search platforms, but not the underlying model architecture. The result, as Black (2021) would note, is decentred regulation—a fragmented ecosystem of overlapping authorities, standards, and private ordering.

Within this ecosystem, IDBs generate regulatory indeterminacy across at least three axes.

First, their dual status as both content and actor confounds platform regulation. IDBs are both content (subject to OSA/DSA moderation) and conversational agents (subject to AIA transparency). The indeterminacy is therefore not exclusivity, but interaction: which obligations are triggered by a particular output or interaction, how responsibilities are allocated between platform and developer, and how overlapping (or conflicting) duties are implemented in practice. Determining which regime applies to a specific interaction is not straightforward. For example, removing illegal content under safety regimes is simpler than deciding if a grief-targeted design constitutes manipulative exploitation, a system-level assessment that eludes standard notice-and-takedown workflows.

Second, their training data engages multiple legal sources, copyrighted material, personal data, and sometimes post-mortem digital remains, each supervised by different regulators. Whether a national data-protection authority, an AI supervisory body, or an intellectual-property office has competence to act depends on how the harm is framed: as a privacy violation, algorithmic manipulation, or copyright infringement.

Third, evidential standards vary. Under Article 5 AIA, providers must avoid subliminal or manipulative techniques that “materially distort behaviour” and cause “significant harm”, or that exploit vulnerabilities linked to age, disability, or a “specific social or economic situation”. As noted above, determining whether bereavement qualifies as such a situation requires normative interpretation and empirical evidence, precisely the conditions of regulatory indeterminacy. Regulators must decide whether grief is an incidental state or a protected vulnerability, an interpretive move that effectively constitutes new law.

Together, these dynamics reveal how IDBs highlight gaps in prevailing paradigms of risk and harm. Traditional product-safety models assume discrete, measurable harms (Yeung and Ranchordas, 2024). And although emerging technologies have historically disrupted established frameworks (Brownsword, Scotford and Yeung, 2017), the cultural, spiritual, and symbolic meanings attached to death infuse these disruptions with an added depth and complexity. Consequently, the harms of IDBs, such as dependency, identity distortion, and exploitation of emotional weakness, are diffuse, relational, and context-dependent. Regulatory mechanisms calibrated for physical or economic risk are ill-suited to govern affective and psychological risk and harm, leaving oversight bodies without clear thresholds or metrics.

#### Institutional Indeterminacy

If IDBs generate indeterminacy by exceeding established categories, regulatory indeterminacy in turn shapes how they are governed. The AI Act’s meta-regulatory design embeds uncertainty by requiring firms to self-audit harms before authorities provide guidance. As Smuha and Yeung (2025) observe, this shifts the locus of regulation from public authority to private governance. For IDBs, whose design directly manipulates emotion and memory, such delegation raises legitimacy and accountability concerns.

In the EU, effective enforcement depends on the interplay between the AI Act, DSA, and national supervisory practices. Article 50 AIA requires providers to label synthetic media, though this may be less helpful for IDBs intended for private use beyond identifying the bot as ‘synthetic’. By contrast, Article 5 targets a narrower category of practices that exploit vulnerability to materially distort behaviour, where regulatory intervention is more intrusive and, in practice, will depend on demonstrating significant harm. Because grief-related harms are cumulative and difficult to quantify, this threshold may operate as a practical barrier to intervention even when manipulation is intuitively harmful.

When IDBs operate within major platforms, platform-level risk duties under the DSA (Art 34) or OSA assume importance. These provisions compel large platforms to assess and mitigate systemic risks to mental health and fundamental rights. However, they address *distributional* rather than *design-level* harms: platforms must moderate outputs, but not necessarily audit the models producing them. This recursive displacement of responsibility allows platforms to point to developers (AIA) while developers point to platform recommender systems.

[The UK’s model intensifies this. Ofcom (2024) confirmed that “virtual clone” bots, including those of the deceased, fall under OSA duties when embedded in user-to-user services. While their outputs are treated as user-generated content, the underlying model’s data provenance and alignment remain outside Ofcom’s remit. Absent a bespoke statute, the UK relies on the distributed capacity of the ICO, CMA, Ofcom, and MHRA to interpret non-binding principles. This regulatory patchwork is further exacerbated by political pressures from tech firms to limit enforcement (Edwards, 2025).

Regulatory indeterminacy is not merely an institutional defect; it is also epistemic. Regulators must act amid uncertainty about the social and psychological effects of IDBs. Empirical evidence on griefbots’ impacts is scarce and contested; risk assessments thus depend heavily on assumptions about human-AI interaction, cultural norms of mourning, and speculative harm. As Ranchordás (2021) argues, anticipatory governance must embrace such epistemic uncertainty while balancing it with proportionality and legitimacy. Over-regulation risks stifling digital afterlife innovation, whereas under-regulation risks normalising exploitation. Furthermore, whether technocratic regulatory bodies are legitimate to adjudicate moral questions about death and dignity remains uncertain.

Ultimately, regulatory indeterminacy is a structural condition. IDBs expose the limits of paradigms that presuppose rational actors and quantifiable risk. The metaphysical dimensions of IDBs create spaces where responsibility remains contested, a feature of the governance environment that demands new institutional capacities to navigate responsibly.

## Conclusion

This paper has sought to bring theoretical clarity to the multiple connections between IDB technology and the notion of indeterminacy. Throughout, we have highlighted how indeterminacy manifests in diverse forms, each raising similar yet distinct challenges for this emerging technology. From a technological perspective, we showed the intrinsic limits of generative and stochastic architectures: how probabilistic modelling, layered fine-tuning and guardrail mechanisms, abstraction and misabstraction, and the epistemic opacity of LLM-based systems produce socio-technical systems whose behaviour can never be fully specified or verified. The social perspective revealed the plurality of meanings and practices through which people engage with the dead via digital interfaces, illustrating how grief, memory, and agency are co-constructed across cultural, emotional, and economic divides. Philosophically, we emphasised the difficulty of defining continuity between the living person and their simulated counterpart, as well as the broader challenge of whether an IDB can ever be meaningfully aligned with its human subject. Legally, we noted that the creation and use of IDBs expose enduring ambiguities in post-mortem rights, harm, dignity, liability and accountability alongside the limits of existing data-protection and personality regimes. At the regulatory level, we showed that fragmented oversight, overlapping mandates, and the absence of clear evidentiary standards produce a field of uncertainty, where discretion and interpretation become central tools of governance.

Together, these analyses demonstrate that IDBs constitute a truly disruptive socio-technical phenomenon, one that unsettles established social and philosophical categories, while testing the resilience of legal and ethical systems that often manage indeterminacy by relying on relatively stable reference points developed over time. We do not suggest that law or ethics were ever designed for a perfectly determinate world. Rather, IDBs intensify and render visible structural tensions that existing frameworks have historically managed through doctrinal stability, institutional settlement, and gradual norm development. Death itself plays a central role in sharpening these dynamics. Unlike other domains of technological disruption, death has no stable or universal meaning; it is saturated with cultural, symbolic, spiritual and moral significance. Bringing these layers to the fore reveals why the indeterminacy surrounding IDBs is deeper and more structurally entrenched. This “specialness” of death places IDBs in a qualitatively different category of socio-technical artefacts, one that resists straightforward integration into existing ethical, legal and regulatory frameworks and exposes their conceptual and normative limits. Recognising and governing through this indeterminacy, rather than attempting to eliminate it, may be the most realistic path to ensuring that these technologies develop in ways that respect plurality, dignity, and the evolving conditions of human remembrance.

By offering a structured analytical map and taxonomy of the different manifestations of indeterminacy in this nascent and unstable field, we hope to stabilise key reference points for future scholarship, which will examine each layer and its core questions in greater empirical and theoretical depth to inform more grounded and context-sensitive approaches to the governance of IDBs.

## Data Availability

Not applicable.

## Footnotes

1
https://www.hereafter.ai/.

2
https://www.storyfile.com/.

3 https://www.myyov.com/.

4 https://www.ae.studio/seanceai.

5 For a more detailed review of the many existing use cases see Kozlovski, 2025 and Buben, 2025.

6 While the use of AI to simulate living individuals is an equally significant and rapidly emerging field, we deliberately exclude it from the present analysis in order to keep the focus of the paper squarely on the distinctive challenges associated with the simulation of the dead.

7 The Anne Clark IDB is inspired by examples discussed both in the literature and in popular media such as the Black Mirror episode “Be Right Back” https://www.imdb.com/title/tt2290780/, and the film ‘Marjorie Prime’ https://www.imdb.com/title/tt4978710/.
